# Diminished Response of Arctic Plants to Warming over Time

**DOI:** 10.1371/journal.pone.0116586

**Published:** 2015-03-13

**Authors:** Kelseyann S. Kremers, Robert D. Hollister, Steven F. Oberbauer

**Affiliations:** 1 Biology, Grand Valley State University, Allendale, MI, United States of America; 2 Biological Sciences, Florida International University, Miami, FL, United States of America; UC Irvine, UNITED STATES

## Abstract

The goal of this study is to determine if the response of arctic plants to warming is consistent across species, locations and time. This study examined the impact of experimental warming and natural temperature variation on plants at Barrow and Atqasuk, Alaska beginning in 1994. We considered observations of plant performance collected from 1994–2000 “short-term” and those from 2007–2012 “long-term”. The plant traits reported are the number of inflorescences, inflorescence height, leaf length, and day of flower emergence. These traits can inform us about larger scale processes such as plant reproductive effort, plant growth, and plant phenology, and therefore provide valuable insight into community dynamics, carbon uptake, and trophic interactions. We categorized traits of all species monitored at each site into temperature response types. We then compared response types across traits, plant growth forms, sites, and over time to analyze the consistency of plant response to warming. Graminoids were the most responsive to warming and showed a positive response to temperature, while shrubs were generally the least responsive. Almost half (49%) of response types (across all traits, species, and sites combined) changed from short-term to long-term. The percent of plants responsive to warming decreased from 57% (short-term) to 46% (long-term). These results indicate that the response of plants to warming varies over time and has diminished overall in recent years.

## Introduction

Higher latitudes show among the greatest and earliest responses to changing climate [1]. Arctic regions have been warming since the mid-1800s, but the warming has accelerated in the past few decades at rates faster than global averages [1]. The harsh climate of the region limits arctic plant growth, and a small increase in temperature may have a great effect on arctic plant communities [2]. Shifts in the composition and abundance of plant species will have important effects on organisms at all trophic levels, as well as many ecosystem processes including nutrient cycling, carbon storage, and solar energy absorption [3]. For example, shifts to a shrub-dominated community can reduce albedo [4, 5, 6], which may impact snowmelt processes and surface radiation budgets [6, 7]. Increased dominance of shrubs and evergreens may also slow nutrient cycling by decreasing soil temperatures, litter quality, and decomposition rates [8]. Understanding how plant communities will change under a changing climate is crucial if we are to gain a full understanding of how ecosystems will change as a whole.

Cold temperatures, low light levels, short growing seasons, and low nutrient availability limit arctic plants [9]. Nitrogen availability is generally considered the primary factor limiting tundra plant production [10, 11]. Temperature and photoperiod can control plant phenology [12, 13]. However, it is likely that no single factor is most limiting across all plant species due to species-specific responses to environmental change [14]. Furthermore, warming may have indirect effects on plant growth that may be more important than the direct effects [9]. Increased temperature may increase decomposition, mineralization, nitrogen fixation, and nutrient absorption [8, 9], changing the way nutrients move through the system. Changes in nutrient cycling will impact arctic plant production and ecosystem carbon storage.

To forecast tundra responses to climate change, many researchers have studied the impact of experimental warming on arctic plants (e.g., [15, 16, 17], and many others). It has become apparent from this work that to detect directional changes in plant responses, long-term studies will be required due to inherent annual variation [18, 19]. Presumably direct effects of temperature on physiology, such as elevated rates of photosynthesis [9] and faster mitotic division [20], dominate short-term responses of plants to temperature. This leads to faster development and often increased growth short-term [13]. It is unlikely that these immediate responses can persist long-term due to the indirect effects of warming on competition and ecosystem properties such as nutrient cycling and trophic interactions [21]. For example, Hollister et al. [22] observed differences between short-term and long-term responses to warming after only 5–7 years of warming. They attributed the decline in response long-term to a shift in the competitive balance of species [22]. It is also possible that initial warming increases nutrient availability and in turn plant productivity, but over time plants grow and store these nutrients in their biomass, making the nutrients less available in the soil [23, 24]. As the nutrients become less available for plant uptake, plant growth will decline [23, 24]. These nutrients will eventually return to the soil through litter decomposition, but the time-scale of nutrient cycling may be much longer depending on shifts in community composition (i.e, shifting from a graminoid-dominated community to a shrub-dominated community, [8]). In contrast to the findings of Hollister et al. [22], several recent studies have found that the long-term and short-term response of vegetation to warming are similar [25, 26, 27].

We analyzed the plots discussed in Hollister et al. [22, 28] to see if there is a difference between the short-term and long-term response of plants to warming after up to 18 years of experimental warming and natural climate variation. We focused on the consistency of plant response to warming across time (short-term vs. long-term), growth forms, locations, and traits. We concentrated on three broad growth forms (graminoids, shrubs, and forbs) and four plant traits (leaf length, inflorescence height, number of inflorescences, and date of flower emergence) at four research sites (differing in latitude and moisture regimes) on the North Slope of Alaska. We chose these broad growth forms because they represent key plant functional types that influence the community and ecosystem in different ways [29]. The plant traits that we selected are also related to large-scale processes and can provide valuable insight into how the community and ecosystem will change with warming. Furthermore, cross-biome syntheses and vegetation models that predict how the Arctic will change under long-term warming frequently use these growth forms and traits, and our goal is to help inform these existing predictions and hypotheses. We hypothesized that plant response to warming would be inconsistent in that: 1) plants would respond differently across growth forms, traits, and sites due to differences in sensitivity to warming, and 2) long-term responses would be less pronounced than short-term responses due to indirect effects of temperature that become more important with increased duration of warming.

## Methods

### Data collection and compilation

**Fig 1:**
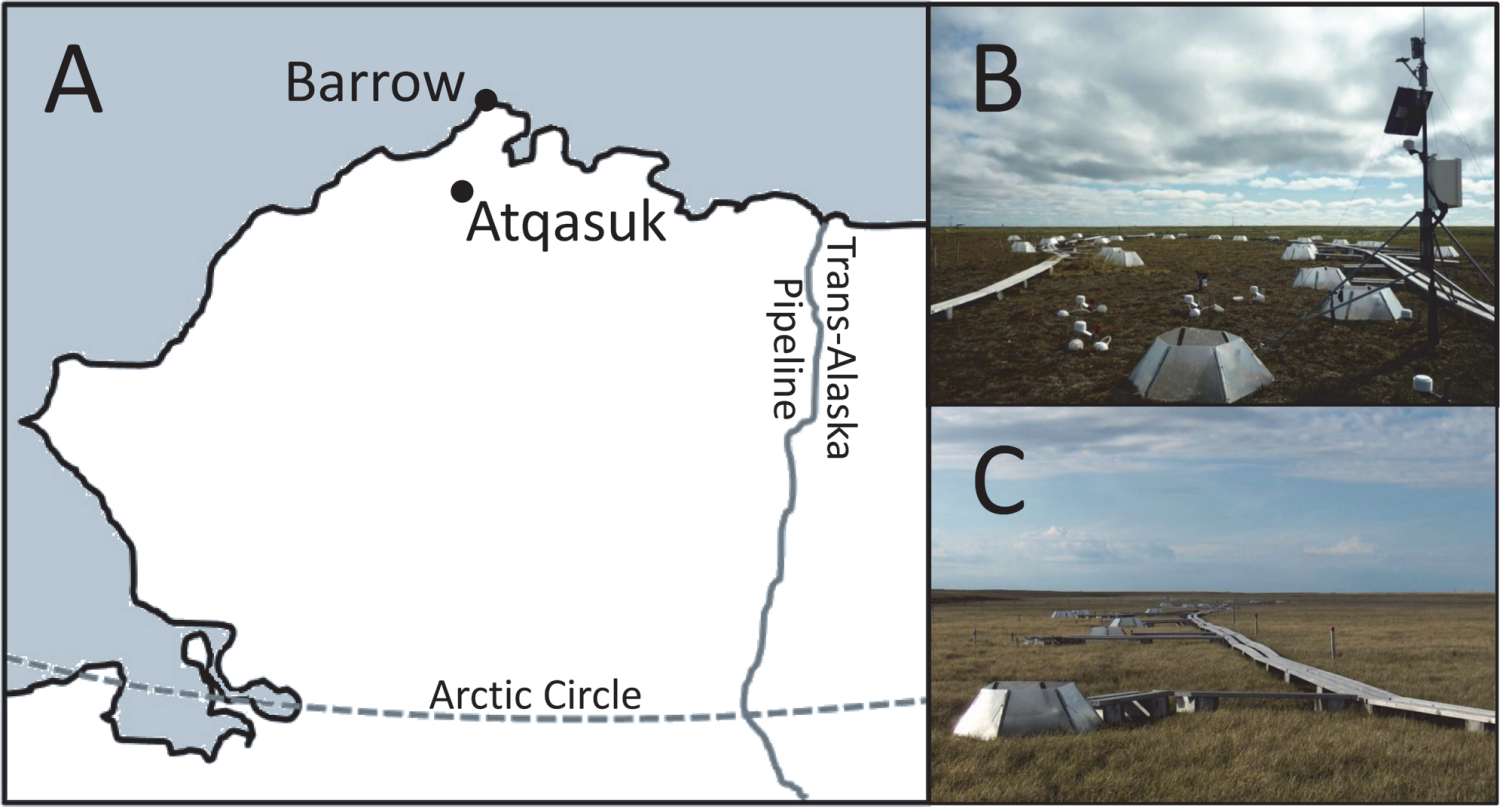
Map of research locations (A), and photographs of Atqasuk dry heath (B) and the Barrow wet meadow (C). Visible in the photographs are the open-top chambers (OTCs) used to increase air temperature and one of the weather stations.

Study sites were established at Barrow (71°19’N, 156°36’W) in 1994 and 1995 and approximately 100 km south at Atqasuk (70°27’N, 157°24’W) in 1996. Mean July temperature is ∼4°C at Barrow and ∼9°C at Atqasuk. Snowmelt occurs in early to mid-June at Barrow and in late May at Atqasuk. Each location contains a dry heath and a wet meadow site (Fig. 1), resulting in a total of four study sites: Barrow dry heath (BD), Barrow wet meadow (BW), Atqasuk dry heath (AD), and Atqasuk wet meadow (AW). Both dry sites (BD and AD) are situated on ridges above thaw lakes. The BD site is dominated by *Cassiope tetragona*, *Salix rotundifolia*, and *Luzula confusa*. The AD site is dominated by *Cassiope tetragona*, *Ledum palustre*, and *Luzula confusa*. Both wet sites (BW and AW) are frequently inundated meadows. The BW site is dominated by *Carex aquatilis*, *Dupontia fisheri*, and *Eriophorum spp*. The AW site is dominated by *Carex aquatilis*, *Eriophorum spp*., and *Salix pulchra*. Lands are privately owned by Ukpeaġvik Iñupiat Corporation and Atqasuk Corporation. The study was approved by North Slope Borough Planning (Permit # NSB 14–786).

The plant canopy was warmed on average 1°C to 3°C over the summer [30] using open-top fiberglass chambers (OTCs, Fig. 1). While these chambers (hexagonal, 35 cm tall, the distance between parallel sides is 103 cm at the base and 60 cm at the top), as with any in-situ experiment, have unintended artifacts (such as decreased wind and light) their additional effects on microclimate and herbivory are documented [30, 31, 32]. OTCs have been shown to be a reasonable analog of a naturally warmer year [33]. Each site contains 24 warmed and 24 control plots. The OTCs have been placed on experimental plots every year after snowmelt since site establishment and removed each fall to prevent snow accumulation, resulting in 16–18 years of experimental warming at the sites. Given that extensive measurements were not conducted in years 2001–2006 (even though the warming treatment was still in place), we used this opportunity to compare approximately the same number of years of observations collected in early years of warming with observations collected in recent years. For this study, we considered the analysis over years 1994–2000 the “short-term” response (ST) to warming and the analysis over years 2007–2012 the “long-term” response (LT).

The traits analyzed in this study (leaf length, inflorescence height, number of inflorescences per plot, and date of flower emergence) were chosen based on their reproducibility across species and on our confidence in the accuracy of the measurements. These traits can also inform us about larger scale processes such as plant reproductive effort, plant growth, and plant phenology and they conform with protocols used for cross biome syntheses [15]. Changes in allocation to plant reproduction (number of inflorescences and inflorescence height) affect plant community structure and function. Changes in growth (leaf length) influence plant productivity and in-turn carbon uptake. Finally, changes in phenology (date of flower emergence) can impact trophic interactions. Within each plot, we monitored all vascular plant species. Leaf length and inflorescence height are measured per individual on the same individuals each year (up to three individuals of each species in each plot), while number of inflorescences and date of flower emergence are measured over the entire plot (per m^2^). We only included species in this analysis if we measured them in seven or more control plots and seven or more warmed plots for a given year in at least three years of both the short-term and long-term range. The number of replicates and traits considered here is more conservative than that reported in Hollister et al. [28]. We did this to maximize confidence that changes observed over time are due to real plant responses and not artifacts of sampling a heterogeneous population.

### Temperature Response Types

Plant temperature response types were characterized according to Hollister et al. [28]. There are six potential temperature response types: Positive dominant (++), positive subordinate (+), negative dominant (--), negative subordinate (-), inconsistent (+/-), and unresponsive (u, Table 1). If a plant trait is dominantly controlled by temperature, then we assume that temperature is the primary factor controlling the response of that trait to warming. If a plant trait is subordinately controlled by temperature, then we assume that factors other than temperature (i.e., nutrients, light) are the primary factors controlling the response of that trait to warming.

**Table 1:** Description of temperatures response types.

Response Type	Symbol	Determination
Unresponsive	u	No significant responses
Positive dominant	++	Significant correlation with TDD, positive warming effect
Positive subordinate	+	Significant response to treatment, no significant correlation with TDD, positive warming effect
Negative dominant	− −	Significant correlation with TDD, negative warming effect
Negative subordinate	−	Significant response to treatment, no significant correlation with TDD, negative warming effect
Inconsistent	+/−	Response to warming was positive, negative, or none depending on the year

Significance values for statistical analyses are given in S1 Table.

We performed the characterization twice, once for the short-term response and once for the long-term response. For each species at a site, we conducted a linear regression relating the plant trait response to the accumulated Thawing Degree Days (TDD, the sum of the average temperature for each day excluding negative temperatures) and a 2-factor repeated measures ANOVA relating response to treatment and year. For date of flower emergence, we calculated TDDs from day of snow melt until the day of flower emergence. For all other traits, we calculated TDDs from day of snow melt until the end of the growing season (August 15^th^). We used TDD from the previous season for correlations with number of inflorescences, as this should be a better predictor than TDD of the current season [34, 35]. If the trait was significantly correlated with TDD, then we considered temperature to be a dominant factor controlling plant response. If the trait responded to experimental warming but was not correlated with TDD, then we considered temperature to be a subordinate factor controlling plant response. We then characterized the response as positive or negative based on the direction of change each year. If the response was positive in some years and negative in others, we characterized the response as inconsistent. If there was no overall correlation with TDD and no significant response to warming treatment, then we considered the trait unresponsive to temperature. Therefore, when we refer to the percent of plants “responsive to warming”, we include plant traits dominantly, subordinately, or inconsistently controlled by temperature. The six possible temperature response types are described in Table 1.

### Statistical Analysis

All statistical analyses described above were performed in R 2.13.1 [36]. Relationships were considered significant if P<0.05. P-values for all statistical tests can be found in S1 Table.

## Results

**Fig 2:**
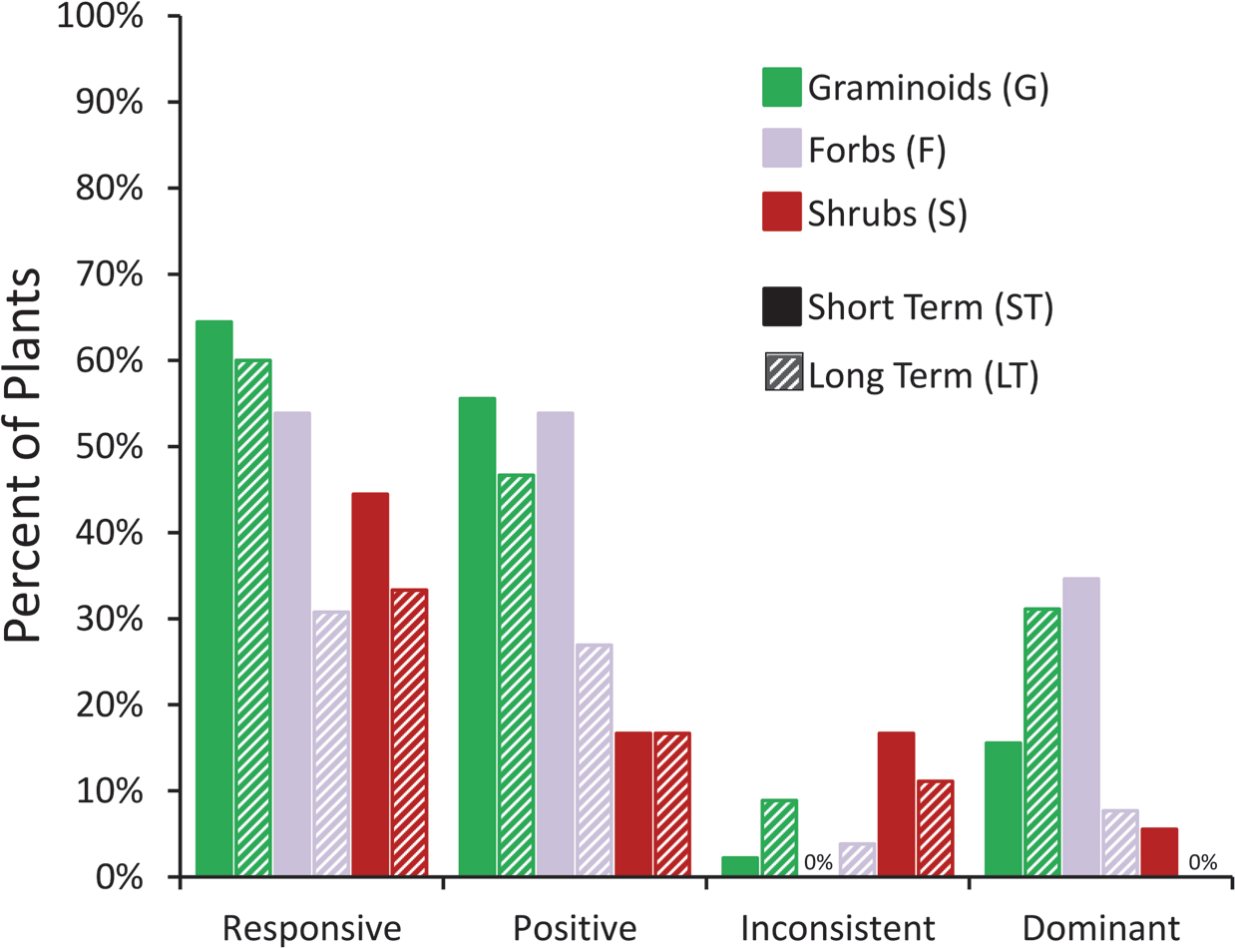
Percent of temperature response types from Table 1 characterized as responsive, positive, dominant, or inconsistent. Short-term (solid bars) and long-term (diagonal lines) observations are included.

**Fig 3:**
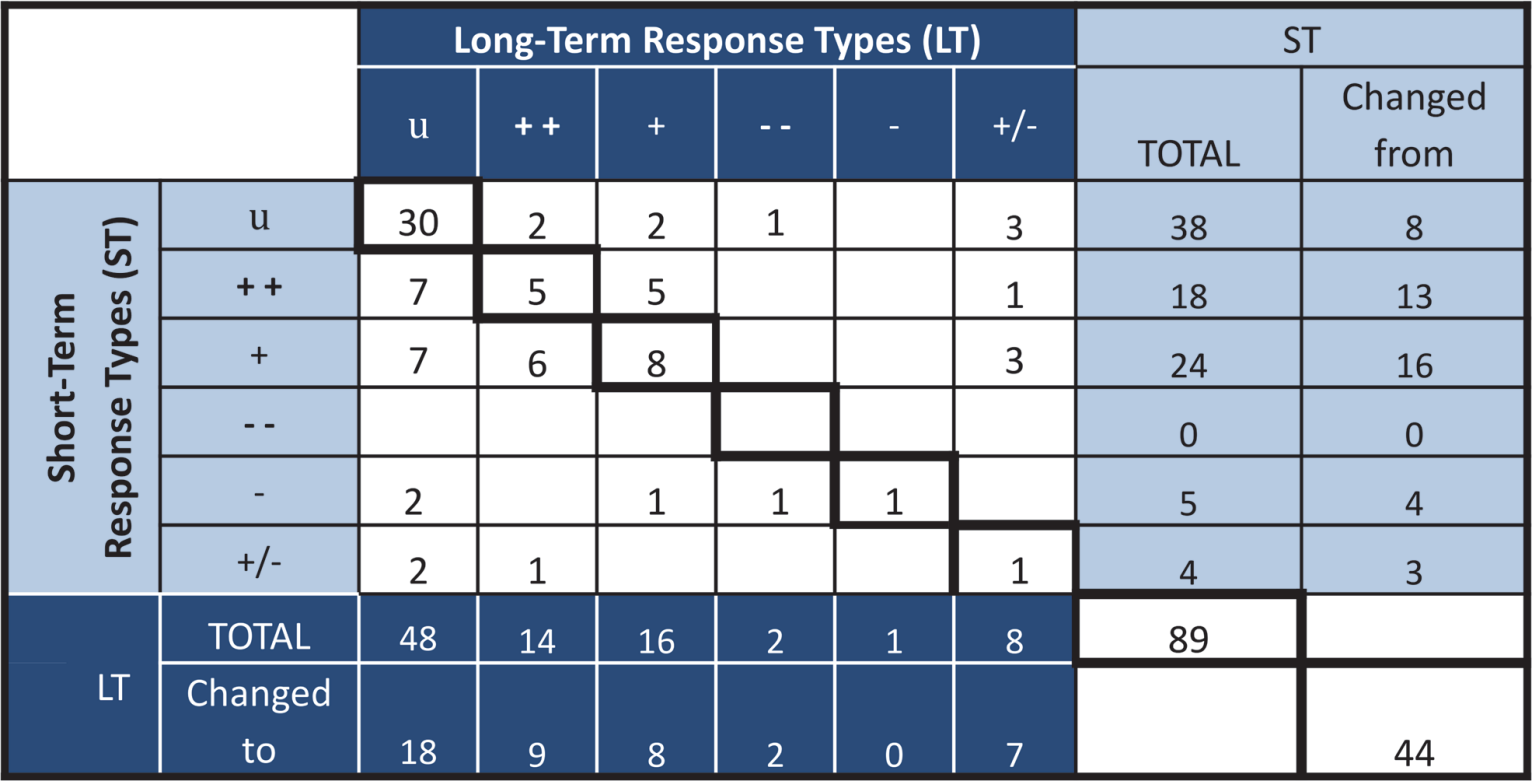
Matrix displaying the change in the sum of temperature response types from short-term to long-term. Calculations include all species, site, and trait combinations in Table 1 for short-term (ST, light blue) and long-term (LT, dark blue). The diagonal (bold box) shows the number of response types that were categorized the same in the short-term and long-term (center) or the sum (bottom right).

When we examined the consistency of the temperature response types of all traits of all monitored species at the four sites, we found 49% (44 occurrences out of 89 reported) of the temperature response types changed between the short-term and long-term observations (Table 2, Fig. 2, Fig. 3); the proportion would be 37% if we did not distinguish between dominant and subordinate response types (++ and + or -- and -). The percentage of plants characterized as responsive to warming (++, +,--,-, or +/-) fell from 57% in the short-term to 46% in the long-term. Of the plants characterized as unresponsive in the short or long-term, only 54% were unresponsive in both. Excluding plants that never responded to warming, a plant was at least two times more likely to become unresponsive than to become responsive in the long-term (Fig. 3).

**Table 2:** Temperature response type characterization of each trait of all monitored species at the four study sites determined from short-term (ST, grey, 1994–2000) and long-term (LT, black, 2007–2012) observations.

	Number of Inflorescences		Inflorescence Height		Leaf Length		Day of Flower Emergence	
	ST	LT	ST	LT	ST	LT	ST	LT
**Atqasuk Dry Heath**								
*Hierochloe alpina* (G)	+	u	+	+	++	+	−	− −
*Luzula arctica* (G)	.	.	+/−	++	u	u	.	.
*Luzula confusa* (G)	u	u	.	.	u	++	+	u
*Polygonum bistorta* (F)	.	.	.	.	++	u	.	.
*Cassiope tetragona* (S)	.	.	.	.	u	u	.	.
*Diapensia lapponica* (S)	−	u	+/−	u	u	u	u	u
*Ledum palustre* (S)	u	u	.	.	++	u	u	+
*Vaccinium vitis-idaea* (S)	−	−	.	.	u	u	u	u
**Atqasuk Wet Meadow**									
*Carex aquatilis* (G)	u	u	+	+	+	+	−	+
*Dupontia fisheri* (G)	.	.	.	.	u	u	.	.
*Eriophorum angustifolium* (G)	−	u	+	+	++	+	.	.
*Eriophorum russeolum* (G)	.	.	.	.	++	u	.	.
*Pedicularis sudetica* (F)	.	.	.	.	u	u	.	.
**Barrow Dry Heath**								
*Arctagrostis latifolia* (G)	.	.	.	.	+	u	.	.
*Luzula arctica* (G)	.	.	.	.	u	+	.	.
*Luzula confusa (G)*	u	u	++	++	+	++	+	+
*Poa arctica* (G)	u	+/−	+	++	+	++	.	.
*Potentilla hyparctica* (F)	u	+/−	++	++	.	.	+	+
*Saxifraga punctata* (F)	u	u	++	u	+	+	u	u
*Senecio atropurpureus* (F)	.	.	.	.	u	u	.	.
*Stellaria laeta* (F)	.	.	.	.	.	.	+	u
*Cassiope tetragona* (S)	.	.	.	.	u	u	+	+/−
*Salix rotundifolia* female (S)	u	+/−	+	++	.	.	+/−	+/−
*Salix rotundifolia* male (S)	u	u	.	.	.	.	+/−	u
**Barrow Wet Meadow**
*Carex aquatilis* (G)	u	− −	++	++	+	++	+	+
*Dupontia fisheri* (G)	u	u	++	++	u	++	+	+/−
*Eriophorum angustifolium* (G)	u	u	u	u	+	u	.	.
*Eriophorum russeolum* (G)	.	.	.	.	u	u	.	.
*Hierochloe pauciflora* (G)	u	u	++	+/−	+	++	+	u
*Luzula arctica* (G)	u	u	.	.	.	.	.	.
*Luzula confusa* (G)	.	.	+	+/−	.	.	.	.
*Cardamine pratensis* (F)	++	u	++	+	++	+	.	.
*Draba lactea* (F)	.	.	++	++	.	.	u	u
*Saxifraga cernua* (F)	u	u	++	u	u	u	u	u
*Saxifraga foliolosa* (F)	u	u	++	+	u	u	.	.
*Saxifraga hieracifolia* (F)	u	u	++	u	.	.	+	u

Notes: Species are organized by site and by broad growth form: graminoids (G), forbs (F), and shrubs (S).Temperature response types used were: unresponsive (u), positively and dominantly controlled (++), positively and subordinately controlled (+), negatively and dominantly controlled (--), negatively and subordinately controlled (-), or inconsistent (+/-). In many cases the trait was not recorded or there was not enough data for a response type to be assigned (.).

When we compared temperature response types by trait (Table 2), the number of inflorescences was the least responsive trait to warming (23% ST, 23% LT), while inflorescence height was the most responsive (95% ST, 75% LT). We did not find any observations dominantly controlled by temperature for the date of flower emergence. Inflorescence height and date of flower emergence showed the greatest changes between short-term and long-term. Both traits showed a decrease in the percent of responsive plants (Inflorescence height: 95% ST, 75% LT; Date of flower emergence: 68% ST, 47% LT) and a decrease in the percent of positive responders (Inflorescence height: 85% ST, 65% LT; Date of flower emergence: 47% ST, 32% LT).

When we compared temperature response types by site (Table 2), the percent of plants that responded to warming decreased from the short-term to the long-term at all sites. At the Atqasuk dry heath, we found only 33% of cases responsive to warming in the long-term, compared to 60%, 42%, and 50% at the Barrow dry heath, Barrow wet meadow, and Atqasuk wet meadow sites, respectively. Atqasuk dry heath had the lowest percentage of cases positively controlled by warming in the long-term (24%), while Atqasuk wet meadow had the highest (50%).

When we compared temperature response types by growth form (Table 2, Fig. 2, Fig. 3), 53% of graminoids, 42% of forbs, and 44% of shrubs changed response types between short-term and long-term. Graminoids were the most responsive to warming (64% ST, 60% LT) and showed the highest percentage of positive responders in both short-term (56%) and long-term (47%). Shrubs were least responsive to warming (44% ST, 33% LT), and had the lowest percent of positive responders (17% for ST and LT). Shrubs also had the highest percent of cases that responded inconsistently from year to year (17% ST, 11% LT). The percent of forb (F) and shrub (S) response types dominantly controlled by temperature decreased in the long-term (F: 35% ST, 8% LT; S: 6% ST, 0% LT), but the percent of dominantly controlled graminoids increased (16% ST, 31% LT). Half of the forb response-types that we considered positive in the short-term became unresponsive or inconsistent in the long-term. Forbs also showed the greatest decrease in the percent of cases responsive to warming (54% ST, 31% LT) and dominantly controlled by warming (34.6% ST, 8% LT) from short-term to long-term (Fig. 2).

## Discussion

Our results show that the response of arctic plants to warming varies with location, time, trait, and growth form, which is consistent with the findings of previous studies [15, 37, 38]. Nearly half of the temperature response types changed between short-term and long-term, indicating that the initial response of arctic plants to warming may not be maintained when warming occurs over many years. This is further supported by our finding that the percent of responsive plants decreased in the long-term, and traits that were responsive in either the short-term or long-term were twice as likely to become unresponsive than to become responsive in the long-term. The response of tundra plants to increased temperature may dampen over time [39]. Other factors that impact plant performance, such as competition and differences between ecotypes, may buffer or obscure warming effects long-term [38].

The dampened response to warming over time may be due to lack of available nutrients as the plants have grown and stored the nutrients in their biomass, making nutrients less available in the soil. Plant productivity responds consistently to nutrient addition across several tundra plant communities, indicating extensive nutrient limitation in the Arctic [10, 11]. Other environmental factors, such as temperature, cause ecosystem level changes only if nutrients become more available [40, 41]. Warming can increase nutrient availability [9], but if these nutrients become temporarily stored in plant biomass due to increased nutrient uptake and growth during the initial short-term response to warming, the nutrients will be less available in the soil to increase plant productivity in the long-term. Competition for these nutrients may increase if the number of individuals is increasing or if plant community composition is changing. Cycling of these nutrients, and therefore their return to the soil through litter decomposition may slow if the plant community changes (i.e, from graminoids with fast turn-over and decomposition to shrubs with slower litterfall and decomposition, [8]). Measurements of foliar nutrients and plant competition for nutrients would be helpful in teasing apart these interactions.

A decrease in available nutrients over time does not, however, explain the decreased response in date of flower emergence. Temperature and photoperiod control plant phenology in the Arctic [12, 13]. After four years of warming, Arft et al. [15] found that flowering phenology was significantly earlier. Our results imply that this response may be less pronounced in the long-term. In non-arctic regions, a decline in response of phenological traits to warming over time may be due to the short photoperiod in the spring. In the long-term, the flowering date may shift so early in the season that the temperature limitation is less than the photoperiod limitation. This could result in a diminished response of phenological traits to warming over time. However, at our arctic sites day length is 24 hours by the time the snow melts, leaving us unsure about the mechanism resulting in a dampened phenological response over time. Plants unable to adjust their phenology with climate change may be more vulnerable [42]. If plants alter their phenology but herbivores or pollinators do not shift their foraging habits to align with this change in phenology, plant reproductive success may change [43]. Thus we need further research to understand the implication of trophic interactions [44, 45]. In addition, earlier leaf phenology may result in depleted foliar nitrogen and phosphorus in some species [29]. It is possible then that earlier phenology results in a feedback mechanism that depletes nutrient availability to plants, which could then further explain the diminished response in growth we observed over time.

It would be very useful to have reliable information on the recruitment and competition between species. This information could give us more insight on community and ecosystem level changes. For instance, the decline in leaf growth over time may not necessarily mean there is a decline in overall productivity. Plants could be producing more individuals rather than increasing growth, which would still result in increased productivity over time. However, trends in a meta-analysis conducted by Elmendorf et al. [37] show a slight increase in the amount of bare ground with increased summer temperature, implying that there may not be an increase in the number of individuals produced with warming. Furthermore, Brooker and van der Wal [46] found that increased biomass due to soil warming was largely driven by increased size rather than an increase in the number of individuals. Our lab is currently making an effort to collect these data in order to better inform this process.

Shrubs were the least responsive to warming and had the fewest positive responders. This finding may seem contradictory to reports of shrub expansion northward with climate change [47, 48]. However, Elmendorf et al. [37] showed that summer warming had a more positive impact on shrubs in locations that were already warm to begin with. This may explain why we observed such weak positive responses of shrubs in our higher arctic study sites. Furthermore, the shrub species presented in this study are evergreen or prostrate, and evergreen and prostrate shrubs did not show a positive response to warming in the meta-analysis conducted by Elmendorf et al. [37]. Hudson and Henry [49] also suggested that the evergreen shrub heath communities in the high arctic show a community-level resistance to long-term experimental warming. Therefore, our results are consistent with previous research, and provide further evidence that species and location may restrict shrub expansion and dominance under warmed conditions.

Graminoids were the most responsive to warming and showed the highest percentage of positive responders in both short-term and long-term. Graminoids responded the most consistently from short-term to long-term, and the percent of graminoids dominantly controlled by temperature increased in the long-term. Studies have shown that graminoids respond strongly to warming and fertilization treatments [16, 48, 50]. Fast-growing graminoids are able to adjust nutrient uptake rates and growth rates under optimal conditions more quickly than slow-growing shrubs [51], which could explain why graminoids show a much stronger response to warming. In other environmental manipulations, graminoids dominate the positive response initially, but within 5–10 years deciduous shrubs typically become dominant [51]. However, as stated previously, the deciduous shrubs that are present at our site do not have a strong temperature response and may be unable to out-compete the graminoids. Therefore, plant communities similar to our research sites may become dominated by graminoids under long-term warming.

Forbs showed the greatest decrease in the percent of plant traits considered responsive to temperature, positively controlled by temperature, and dominantly controlled by temperature from short-term to long-term. The decline in these response-types long-term suggests that forbs may acclimate to long-term warming better than shrubs and graminoids. Other studies have also found that forbs do not respond significantly to warming or nutrient additions [16, 50, 52]. Forbs differ from graminoids by having broader leaves and by dispersing mainly through insect pollination rather than wind or clonally [29]. Most of the forbs in this study are dicots, which may be more sensitive to changes in light availability than to changes in temperature, nutrient availability, or soil moisture [16]. Differences in leaf structure, dispersal mechanisms, and sensitivity to environmental change could explain why forbs are less responsive to warming than other non-woody plants, namely graminoids. A community that has many forb species or a high abundance of forbs may be more resistant to changes in temperature or nutrients. It is important to note, however, that forbs may be indirectly impacted by warming and increased nutrient availability if graminoids and shrubs grow enough to shade-out the understory forb species.

The Atqasuk dry heath had the fewest temperature response types considered positive in the long-term, while the Atqasuk wet meadow had the greatest. Previous studies have shown that plant responses to a warmer, drier climate may vary within a landscape [38]. Furthermore, research has suggested that plant response to warming may be site-specific [22, 37, 49]. However, it is important to note that Atqasuk wet meadow has the fewest number of species compared to the other sites, and all but one of the species analyzed are graminoids. Given graminoids showed the most positive response to warming, the effect observed at Atqasuk wet meadow may have been due to the plant species present in that community rather than the abiotic characteristics of the site. Also, Atqasuk dry heath is dry and sandy, so the increased temperature could be amplifying that dryness and negatively impacting plants at that site. The Barrow Wet site showed the greatest decrease in positive responders to warming, and both Barrow Dry and Barrow Wet had the greatest percent of inconsistent responders in the long-term. These sites have more forb species than the Atqasuk sites. Neither of the Barrow sites have a high abundance of tall plants that would shade-out the forbs, even after warming treatment. Given that forbs may be resistant to long term warming, the lack of response long-term at these sites could be partly due to the abundance of forbs.

This study shows that the response of a plant trait may vary over time and that the most common pattern at these four sites is for the magnitude of response to warming to diminish over time. While the traits examined here are not necessarily the best suited to predict the long-term success of the species, they do provide insights on the pattern of plant response. When preparing models to predict changes in tundra plant communities with climate change, it is important to consider the variability of plant response to climate change over space and time. We have shown that the long-term response of plants to warming may differ from the short-term response. If we base our predictions of the long-term impacts of climate change on what we see in the short-term, our predictions may be incorrect. Therefore, coordinated long-term monitoring studies are necessary to accurately document, understand, and predict vegetation change over time [53, 54].

## Supporting Information

S1 Table P values for statistical tests used to assign temperature response types for both short-term (ST) and long-term (LT). (DOCX)
