# Supplementary material for: Diminished Response of Arctic Plants to Warming over Time
Source: PLoS One. 2015 Mar 13;10(3):e0116586. doi: 10.1371/journal.pone.0116586 (PMC4358989; doi:10.1371/journal.pone.0116586)
Supplement: S1 Table — (DOCX) [file pone.0116586.s001.docx]

Table S1: P values for statistical tests used to assign temperature response types for both short-term (ST) and long-term (LT). Regression TDD represents the linear regression relating the plant trait response to the accumulated thawing degree day (TDD) of the summer. The ANOVA values are from the 2-factor repeated measures ANOVA relating response to treatment and year, where ANOVA Treatment is the response to experimental warming, ANOVA Year is the year response, and ANOVA Treatment*Year is the interaction. The sites are Atqasuk wet meadow (AW), Atqasuk dry heath (AD), Barrow wet meadow (BW), and Barrow dry heath (BD).

| **Trait** | **Time** | **Site** | **Species** | **Regression**  **TDD** | **ANOVA Treatment** | **ANOVA Year** | **ANOVA Treatment*Year** |
| --- | --- | --- | --- | --- | --- | --- | --- |
| Flower Emergence | ST | AW | *C. aquatilis* | <0.05 | 0.36 | <0.05 | <0.05 |
| Flower Emergence | ST | BW | *C. aquatilis* | 0.99 | <0.05 | <0.05 | 0.44 |
| Flower Emergence | ST | BD | *C. tetragona* | 0.27 | <0.05 | <0.05 | <0.05 |
| Flower Emergence | ST | BW | *D. fisheri* | 0.17 | <0.05 | <0.05 | 0.11 |
| Flower Emergence | ST | BW | *D. lactaea* | 0.67 | 0.27 | <0.05 | 0.8 |
| Flower Emergence | ST | AD | *D. lapponica* | 0.5 | 0.3 | <0.05 | 0.14 |
| Flower Emergence | ST | AD | *H. alpina* | <0.05 | <0.05 | <0.05 | <0.05 |
| Flower Emergence | ST | BW | *H. pauciflora* | 0.37 | <0.05 | <0.05 | 0.23 |
| Flower Emergence | ST | AD | *L. confusa* | 0.11 | <0.05 | <0.05 | 0.11 |
| Flower Emergence | ST | BD | *L. confusa* | 0.98 | <0.05 | <0.05 | <0.05 |
| Flower Emergence | ST | AD | *L. palustre* | 0.69 | 0.51 | <0.05 | 0.87 |
| Flower Emergence | ST | BD | *P. hyparctica* | 0.92 | <0.05 | <0.05 | <0.05 |
| Flower Emergence | ST | BW | *S. cernua* | 0.6 | 0.25 | <0.05 | 0.54 |
| Flower Emergence | ST | BW | *S. hieracifolia* | 0.65 | <0.05 | <0.05 | 0.87 |
| Flower Emergence | ST | BD | *S. laeta* | 0.8 | <0.05 | <0.05 | 0.37 |
| Flower Emergence | ST | BD | *S. punctata* | 0.36 | 0.09 | <0.05 | 0.69 |
| Flower Emergence | ST | BD | *S. rotundifolia* (F) | 0.54 | <0.05 | <0.05 | <0.05 |
| Flower Emergence | ST | BD | *S. rotundifolia* (M) | 0.64 | <0.05 | <0.05 | <0.05 |
| Flower Emergence | ST | AD | *V. vitis-idaea* | 0.8 | 0.7 | <0.05 | 0.58 |
| Flower Emergence | LT | AW | *C. aquatilis* | 0.68 | <0.05 | <0.05 | <0.05 |
| Flower Emergence | LT | BW | *C. aquatilis* | 0.67 | <0.05 | <0.05 | 0.08 |
| Flower Emergence | LT | BD | *C. tetragona* | 0.65 | <0.05 | <0.05 | <0.05 |
| Flower Emergence | LT | BW | *D. fisheri* | 0.71 | <0.05 | <0.05 | 0.13 |
| Flower Emergence | LT | BW | *D. lactaea* | 0.86 | 0.67 | <0.05 | 0.99 |
| Flower Emergence | LT | AD | *D. lapponica* | 0.16 | 0.98 | <0.05 | 0.26 |
| Flower Emergence | LT | AD | *H. alpina* | <0.05 | 0.13 | <0.05 | 0.26 |
| Flower Emergence | LT | BW | *H. pauciflora* | 0.61 | 0.22 | <0.05 | 0.59 |
| Flower Emergence | LT | AD | *L. confusa* | 0.09 | 0.62 | <0.05 | 0.09 |
| Flower Emergence | LT | BD | *L. confusa* | 0.84 | <0.05 | <0.05 | 0.27 |
| Flower Emergence | LT | AD | *L. palustre* | 0.91 | <0.05 | <0.05 | 0.15 |
| Flower Emergence | LT | BD | *P. hyparctica* | 0.93 | <0.05 | <0.05 | <0.05 |
| Flower Emergence | LT | BW | *S. cernua* | 0.75 | 0.91 | <0.05 | 0.62 |
| Flower Emergence | LT | BW | *S. hieracifolia* | 0.74 | 0.12 | <0.05 | <0.05 |
| Flower Emergence | LT | BD | *S. laeta* | 0.63 | 0.3 | <0.05 | 0.43 |
| Flower Emergence | LT | BD | *S. punctata* | 0.79 | 0.16 | <0.05 | 0.23 |
| Flower Emergence | LT | BD | *S. rotundifolia* (F) | 0.63 | <0.05 | <0.05 | 0.42 |
| Flower Emergence | LT | BD | *S. rotundifolia* (M) | 0.74 | 0.23 | <0.05 | 0.1 |
| Flower Emergence | LT | AD | *V. vitis-idaea* | 0.08 | 0.95 | <0.05 | <0.05 |
| Number Inflor. | ST | AW | *C. aquatilis* | 0.20 | 0.25 | < 0.05 | < 0.05 |
| Number Inflor. | ST | BW | *C. aquatilis* | < 0.05 | 0.40 | < 0.05 | 0.78 |
| Number Inflor. | ST | BW | *C. pratensis* | 0.06 | < 0.05 | < 0.05 | 0.17 |
| Number Inflor. | ST | BW | *D. fisheri* | 0.05 | 0.05 | < 0.05 | < 0.05 |
| Number Inflor. | ST | AD | *D. lapponica* | 0.47 | < 0.05 | < 0.05 | 0.90 |
| Number Inflor. | ST | AW | *E. angustifolia* | 0.48 | < 0.05 | 0.08 | 0.41 |
| Number Inflor. | ST | BW | *E. angustifolia* | 0.48 | 0.71 | < 0.05 | 0.87 |
| Number Inflor. | ST | AD | *H. alpina* | 0.06 | < 0.05 | < 0.05 | 0.81 |
| Number Inflor. | ST | BW | *H. pauciflora* | 0.55 | 0.71 | < 0.05 | 0.11 |
| Number Inflor. | ST | BW | *L. arctica* | 0.41 | 0.97 | 0.92 | 0.64 |
| Number Inflor. | ST | AD | *L. confusa* | 0.69 | 0.37 | < 0.05 | 0.09 |
| Number Inflor. | ST | BD | *L. confusa* | 0.41 | 0.22 | < 0.05 | 0.08 |
| Number Inflor. | ST | AD | *L. palustre* | 0.13 | 0.06 | < 0.05 | 0.12 |
| Number Inflor. | ST | BD | *P. arctica* | 0.21 | 0.42 | < 0.05 | 0.53 |
| Number Inflor. | ST | BD | *P. hyparctica* | 0.60 | 0.76 | < 0.05 | 0.06 |
| Number Inflor. | ST | BW | *S. cernua* | 0.10 | 0.94 | < 0.05 | 0.73 |
| Number Inflor. | ST | BW | *S. foliolosa* | 0.50 | 0.93 | < 0.05 | 0.33 |
| Number Inflor. | ST | BW | *S. hieracifolia* | 0.98 | 0.75 | < 0.05 | 0.99 |
| Number Inflor. | ST | BD | *S. punctata* | 0.50 | 0.38 | < 0.05 | 0.18 |
| Number Inflor. | ST | BD | *S. rotundifolia* (F) | 0.18 | 0.49 | < 0.05 | < 0.05 |
| Number Inflor. | ST | BD | *S. rotundifolia* (M) | 0.21 | 0.71 | < 0.05 | 0.24 |
| Number Inflor. | ST | AD | *V. vitis-idaea* | 0.63 | < 0.05 | < 0.05 | < 0.05 |
| Number Inflor. | LT | AW | *C. aquatilis* | 0.47 | 0.76 | < 0.05 | 0.63 |
| Number Inflor. | LT | BW | *C. aquatilis* | 0.16 | 0.05 | < 0.05 | < 0.05 |
| Number Inflor. | LT | BW | *C. pratensis* | 0.98 | 0.10 | < 0.05 | 0.47 |
| Number Inflor. | LT | BW | *D. fisheri* | < 0.05 | 0.06 | < 0.05 | < 0.05 |
| Number Inflor. | LT | AD | *D. lapponica* | 0.73 | 0.53 | < 0.05 | 0.94 |
| Number Inflor. | LT | AW | *E. angustifolia* | 0.90 | 0.63 | < 0.05 | 0.44 |
| Number Inflor. | LT | BW | *E. angustifolia* | 0.52 | 0.13 | < 0.05 | 0.96 |
| Number Inflor. | LT | AD | *H. alpina* | 0.06 | 0.81 | < 0.05 | 0.50 |
| Number Inflor. | LT | BW | *H. pauciflora* | 0.37 | 0.08 | < 0.05 | 0.08 |
| Number Inflor. | LT | BW | *L. arctica* | 0.54 | 0.70 | < 0.05 | 0.69 |
| Number Inflor. | LT | AD | *L. confusa* | 0.96 | 0.77 | < 0.05 | 0.48 |
| Number Inflor. | LT | BD | *L. confusa* | 0.44 | 0.74 | < 0.05 | 0.82 |
| Number Inflor. | LT | AD | *L. palustre* | 0.17 | 0.27 | < 0.05 | 0.06 |
| Number Inflor. | LT | BD | *P. arctica* | < 0.05 | 0.11 | < 0.05 | 0.33 |
| Number Inflor. | LT | BD | *P. hyparctica* | < 0.05 | 0.27 | < 0.05 | < 0.05 |
| Number Inflor. | LT | BW | *S. cernua* | 0.69 | 0.21 | < 0.05 | < 0.05 |
| Number Inflor. | LT | BW | *S. foliolosa* | 0.81 | 0.87 | < 0.05 | 0.11 |
| Number Inflor. | LT | BW | *S. hieracifolia* | 0.60 | 0.85 | < 0.05 | 0.66 |
| Number Inflor. | LT | BD | *S. punctata* | 0.06 | 0.39 | < 0.05 | 0.23 |
| Number Inflor. | LT | BD | *S. rotundifolia* (F) | 0.98 | < 0.05 | < 0.05 | < 0.05 |
| Number Inflor. | LT | BD | *S. rotundifolia* (M) | 1.00 | 0.96 | < 0.05 | < 0.05 |
| Number Inflor. | LT | AD | *V. vitis-idaea* | 0.80 | < 0.05 | < 0.05 | 0.17 |
| Inflor. Ht. | ST | AW | *C. aquatilis* | 0.44 | <0.05 | <0.05 | <0.05 |
| Inflor. Ht. | ST | BW | *C. aquatilis* | <0.05 | <0.05 | <0.05 | 0.45 |
| Inflor. Ht. | ST | BW | *C. pratensis* | <0.05 | <0.05 | <0.05 | 0.06 |
| Inflor. Ht. | ST | BW | *D. fisheri* | <0.05 | 0.83 | <0.05 | 0.5 |
| Inflor. Ht. | ST | BW | *D. lactaea* | <0.05 | <0.05 | <0.05 | 0.08 |
| Inflor. Ht. | ST | AD | *D. lapponica* | 0.59 | <0.05 | <0.05 | 0.11 |
| Inflor. Ht. | ST | AW | *E. angustifolia* | 0.41 | <0.05 | <0.05 | 0.07 |
| Inflor. Ht. | ST | BW | *E. angustifolia* | 0.62 | 0.1 | <0.05 | <0.05 |
| Inflor. Ht. | ST | AD | *H. alpina* | 0.15 | <0.05 | <0.05 | <0.05 |
| Inflor. Ht. | ST | BW | *H. pauciflora* | <0.05 | <0.05 | <0.05 | 0.42 |
| Inflor. Ht. | ST | AD | *L. confusa* | 0.91 | <0.05 | <0.05 | <0.05 |
| Inflor. Ht. | ST | BD | *L. confusa* | <0.05 | <0.05 | <0.05 | <0.05 |
| Inflor. Ht. | ST | BW | *L. confusa* | 0.09 | <0.05 | <0.05 | 0.37 |
| Inflor. Ht. | ST | BD | *P. arctica* | 0.08 | <0.05 | <0.05 | 0.45 |
| Inflor. Ht. | ST | BD | *P. hyparctica* | <0.05 | 0.13 | <0.05 | <0.05 |
| Inflor. Ht. | ST | BW | *S. cernua* | <0.05 | <0.05 | <0.05 | 0.48 |
| Inflor. Ht. | ST | BW | *S. foliolosa* | <0.05 | <0.05 | <0.05 | 0.78 |
| Inflor. Ht. | ST | BW | *S. hieracifolia* | <0.05 | <0.05 | <0.05 | 0.54 |
| Inflor. Ht. | ST | BD | *S. punctata* | <0.05 | <0.05 | <0.05 | 0.44 |
| Inflor. Ht. | ST | BD | *S. rotundifolia* | 0.25 | <0.05 | <0.05 | 0.11 |
| Inflor. Ht. | LT | AW | *C. aquatilis* | 0.22 | <0.05 | <0.05 | 0.23 |
| Inflor. Ht. | LT | BW | *C. aquatilis* | <0.05 | <0.05 | <0.05 | <0.05 |
| Inflor. Ht. | LT | BW | *C. pratensis* | 0.1 | <0.05 | <0.05 | <0.05 |
| Inflor. Ht. | LT | BW | *D. fisheri* | <0.05 | <0.05 | <0.05 | 0.16 |
| Inflor. Ht. | LT | BW | *D. lactaea* | <0.05 | <0.05 | 0.57 | 0.62 |
| Inflor. Ht. | LT | AD | *D. lapponica* | 0.45 | 0.82 | <0.05 | 0.19 |
| Inflor. Ht. | LT | AW | *E. angustifolia* | 0.33 | <0.05 | <0.05 | 0.63 |
| Inflor. Ht. | LT | BW | *E. angustifolia* | 0.1 | 0.72 | <0.05 | <0.05 |
| Inflor. Ht. | LT | AD | *H. alpina* | 0.15 | <0.05 | <0.05 | 0.95 |
| Inflor. Ht. | LT | BW | *H. pauciflora* | 0.24 | <0.05 | <0.05 | <0.05 |
| Inflor. Ht. | LT | AD | *L. confusa* | <0.05 | 0.47 | <0.05 | <0.05 |
| Inflor. Ht. | LT | BD | *L. confusa* | <0.05 | <0.05 | <0.05 | 0.93 |
| Inflor. Ht. | LT | BW | *L. confusa* | 0.09 | <0.05 | <0.05 | 0.88 |
| Inflor. Ht. | LT | BD | *P. arctica* | <0.05 | <0.05 | <0.05 | 0.24 |
| Inflor. Ht. | LT | BD | *P. hyparctica* | <0.05 | <0.05 | <0.05 | 0.1 |
| Inflor. Ht. | LT | BW | *S. cernua* | 0.08 | 0.05 | <0.05 | 0.05 |
| Inflor. Ht. | LT | BW | *S. foliolosa* | 0.07 | <0.05 | <0.05 | 0.1 |
| Inflor. Ht. | LT | BW | *S. hieracifolia* | 0.48 | 0.11 | 0.54 | <0.05 |
| Inflor. Ht. | LT | BD | *S. punctata* | 0.27 | 0.16 | <0.05 | <0.05 |
| Inflor. Ht. | LT | BD | *S. rotundifolia* | 0.45 | <0.05 | <0.05 | 0.61 |
| Leaf Length | ST | BD | *A. latifolia* | 0.12 | <0.05 | <0.05 | 0.38 |
| Leaf Length | ST | AW | *C. aquatilis* | 0.1 | <0.05 | <0.05 | 0.78 |
| Leaf Length | ST | BW | *C. aquatilis* | 0.05 | <0.05 | <0.05 | 0.2 |
| Leaf Length | ST | BW | *C. pratensis* | <0.05 | <0.05 | <0.05 | 0.61 |
| Leaf Length | ST | AD | *C. tetragona* | 0.95 | 0.61 | <0.05 | 0.15 |
| Leaf Length | ST | BD | *C. tetragona* | 0.71 | 0.1 | <0.05 | 0.45 |
| Leaf Length | ST | AW | *D. fisheri* | 0.08 | 0.16 | <0.05 | 0.19 |
| Leaf Length | ST | BW | *D. fisheri* | 0.11 | 0.08 | <0.05 | 0.34 |
| Leaf Length | ST | AD | *D. lapponica* | 0.43 | 0.26 | <0.05 | 0.9 |
| Leaf Length | ST | AW | *E. angustifolia* | <0.05 | 0.05 | <0.05 | 0.3 |
| Leaf Length | ST | BW | *E. angustifolia* | 0.24 | 0.22 | <0.05 | 0.56 |
| Leaf Length | ST | AW | *E. russeolum* | <0.05 | <0.05 | <0.05 | 0.13 |
| Leaf Length | ST | BW | *E. russeolum* | 0.07 | <0.05 | <0.05 | 0.7 |
| Leaf Length | ST | AD | *H. alpina* | <0.05 | <0.05 | <0.05 | 0.78 |
| Leaf Length | ST | BW | *H. pauciflora* | 0.07 | <0.05 | <0.05 | 0.11 |
| Leaf Length | ST | AD | *L. arctica* | 0.16 | 0.71 | <0.05 | 0.6 |
| Leaf Length | ST | BD | *L. arctica* | 0.91 | 0.13 | <0.05 | <0.05 |
| Leaf Length | ST | AD | *L. confusa* | 0.08 | 0.37 | <0.05 | 0.05 |
| Leaf Length | ST | BD | *L. confusa* | 0.09 | <0.05 | <0.05 | 0.4 |
| Leaf Length | ST | AD | *L. palustre* | 0.05 | <0.05 | 0.29 | 0.68 |
| Leaf Length | ST | BD | *P. arctica* | 0.14 | <0.05 | <0.05 | 0.86 |
| Leaf Length | ST | AD | *P. bistorta* | <0.05 | 0.27 | <0.05 | 0.47 |
| Leaf Length | ST | AW | *P. sudetica* | 0.12 | 0.09 | <0.05 | 0.26 |
| Leaf Length | ST | BD | *S. atropurpureus* | 0.99 | 0.84 | <0.05 | 0.92 |
| Leaf Length | ST | BW | *S. cernua* | 0.47 | 0.64 | <0.05 | <0.05 |
| Leaf Length | ST | BW | *S. foliolosa* | 0.83 | 0.35 | <0.05 | 0.18 |
| Leaf Length | ST | BD | *S. punctata* | 0.71 | <0.05 | <0.05 | 0.55 |
| Leaf Length | ST | AD | *V. vitis-idaea* | 0.75 | 0.89 | <0.05 | 0.24 |
| Leaf Length | LT | BD | *A. latifolia* | 0.21 | 0.06 | 0.24 | 0.42 |
| Leaf Length | LT | AW | *C. aquatilis* | 0.35 | <0.05 | <0.05 | 0.83 |
| Leaf Length | LT | BW | *C. aquatilis* | <0.05 | <0.05 | <0.05 | 0.41 |
| Leaf Length | LT | BW | *C. pratensis* | 0.64 | <0.05 | <0.05 | 0.15 |
| Leaf Length | LT | AD | *C. tetragona* | 0.74 | 0.61 | <0.05 | 0.64 |
| Leaf Length | LT | BD | *C. tetragona* | 0.25 | 0.66 | <0.05 | 0.54 |
| Leaf Length | LT | AW | *D. fisheri* | 0.45 | 0.13 | <0.05 | 0.07 |
| Leaf Length | LT | BW | *D. fisheri* | <0.05 | <0.05 | <0.05 | 0.14 |
| Leaf Length | LT | AD | *D. lapponica* | 0.08 | 0.73 | <0.05 | 0.59 |
| Leaf Length | LT | AW | *E. angustifolia* | 0.11 | <0.05 | <0.05 | <0.05 |
| Leaf Length | LT | BW | *E. angustifolia* | 0.1 | 0.07 | <0.05 | 0.09 |
| Leaf Length | LT | AW | *E. russeolum* | 0.38 | 0.23 | <0.05 | 0.91 |
| Leaf Length | LT | BW | *E. russeolum* | 0.11 | 0.24 | <0.05 | 0.61 |
| Leaf Length | LT | AD | *H. alpina* | 0.08 | <0.05 | 0.07 | 0.87 |
| Leaf Length | LT | BW | *H. pauciflora* | <0.05 | <0.05 | <0.05 | 0.14 |
| Leaf Length | LT | AD | *L. arctica* | 0.67 | 0.37 | <0.05 | 0.59 |
| Leaf Length | LT | BD | *L. arctica* | 0.09 | <0.05 | <0.05 | 0.51 |
| Leaf Length | LT | AD | *L. confusa* | <0.05 | <0.05 | <0.05 | 0.84 |
| Leaf Length | LT | BD | *L. confusa* | <0.05 | <0.05 | <0.05 | 0.27 |
| Leaf Length | LT | AD | *L. palustre* | 0.54 | 0.31 | <0.05 | 0.86 |
| Leaf Length | LT | BD | *P. arctica* | <0.05 | <0.05 | 0.2 | 0.77 |
| Leaf Length | LT | AD | *P. bistorta* | 0.36 | 0.67 | <0.05 | 0.44 |
| Leaf Length | LT | AW | *P. sudetica* | 0.38 | 0.97 | <0.05 | 0.56 |
| Leaf Length | LT | BD | *S. atropurpureus* | 0.81 | 0.41 | <0.05 | 1 |
| Leaf Length | LT | BW | *S. cernua* | 0.53 | 0.85 | 0.08 | 0.25 |
| Leaf Length | LT | BW | *S. foliolosa* | 0.24 | 0.27 | <0.05 | 0.98 |
| Leaf Length | LT | BD | *S. punctata* | 0.73 | <0.05 | <0.05 | 0.33 |
| Leaf Length | LT | AD | *V. vitis-idaea* | 0.15 | 0.8 | <0.05 | 0.17 |
